# A Sensorimotor Framework for the Neurorehabilitation of Oculomotor Dysfunction in Parkinson’s Disease

**DOI:** 10.3390/jcm15124639

**Published:** 2026-06-15

**Authors:** Tiong Peng Yap

**Affiliations:** 1 IGARD Vision Therapy Center, Singapore 229469, Singapore; tiongyap@igard.com.sg; 2 Institute of Epidemiology and Health Care, University College London, London WC1E 6BT, UK

**Keywords:** Parkinson’s disease, oculomotor dysfunction, convergence insufficiency, vestibular dysfunction, neuro-optometric rehabilitation therapy

## Abstract

Oculomotor dysfunction is an eye movement disorder frequently experienced in patients with Parkinson’s disease. Many patients tend to experience visual symptoms, and this can exacerbate cognitive symptoms when visual tasks become more demanding. The purpose of this review is to characterize oculomotor dysfunction in patients with Parkinson’s disease based on the distinct types of ocular motor deficits and their corresponding impact on the patient’s symptoms, visual perception, activities of daily living, and quality-of-life. A systematic literature search was conducted to identify relevant articles. The results from the sensorimotor framework analysis are categorized in five domains: visual-sensory, visual-motor, visual-perceptual, cognitive processing, and psychosocial challenges. The findings suggest that clinical evaluation and neurorehabilitation should move beyond speed-dependent metrics, but focus on specific non-speed-dependent ocular motor deficits. By fostering interdisciplinary collaboration, healthcare professionals can take proactive steps to address the vision-related challenges faced by patients with Parkinson’s disease.

## 1. Introduction

Parkinson’s disease (PD) is a progressive neurodegenerative disorder characterized by tremors, rigidity, and slow movement (bradykinesia). These motor disorders usually present unilaterally in their early stages before affecting both sides of the body when the conditions advance, and this can impact the activities of daily living where even simple tasks like writing, buttoning clothes, or walking can become arduous. However, eye movement disorders are often overlooked during their early stages as a routine eye examination of a patient’s visual acuity often shows normal results, and the subtle decline in contrast sensitivity and color vision are often missed [1,2,3].

Oculomotor dysfunction, or ocular motor dysfunction, refers to a neurological disorder of eye movement control and coordination, a condition that is frequently observed in patients with PD [4]. This condition encompasses specific ocular motor deficits in relation to the execution of saccadic eye movement, pursuit eye movement, vergence, accommodation, and their integration with the eye’s fixational system and the body’s vestibular system [4]. Each of these neural elements can be clinically evaluated using a neuro-optometric approach [4], and this is distinctively different from oculomotor disorders arising from cranial nerve palsies, such as third (oculomotor) cranial nerve palsy, as the latter are not usually observed in PD and are managed differently.

In Parkinson’s disease, the fronto-striatal and parietal network deficits underlying oculomotor dysfunction is believed to arise primarily from progressive axonal degeneration and dopamine depletion, although this process also involves alterations in multiple non-dopaminergic systems, including cholinergic, GABAergic, and glutamatergic pathways [5,6]. Symptoms are frequently experienced, as nearly 43% of patients have complaints about their vision [7], ranging from mild symptoms, such as eyestrain (asthenopia) and visual fatigue [8,9], to more severe symptoms, such as double vision (diplopia) and headaches [8]. These symptoms tend to mimic a traumatic brain injury (TBI) and concussion due to overlapping neuroanatomical changes in the brainstem, cerebellum, basal ganglia, and frontal lobe [4], even though TBI outcomes tend to be variable and moderated by other factors, such as age, cognitive reserve, and post-traumatic depression. However, it is not possible to distinguish between dopaminergic depletion and intrinsic non-dopaminergic oculomotor dysfunction based on clinical presentation alone. Hence, it is vital to examine each neural element of oculomotor dysfunction carefully in order to plan the rehabilitation effectively.

Given that the mapping of the precise contribution of these neural elements is vital for designing effective neurorehabilitation strategies, the purpose of this review is to characterize oculomotor dysfunction in patients with PD based on the distinct types of ocular motor deficits and their corresponding impact on the patient’s symptoms, visual perception, activities of daily living, and quality-of-life.

## 2. Framework Synthesis and Literature Search Strategy

A systematic literature search was conducted using the PubMed/MEDLINE database for peer-reviewed articles published in English from 2000 to 2026 to capture the sensory, motor, perceptual, cognitive, and psychosocial dimensions of the sensorimotor framework analysis (Section 3). Additionally, a narrative review was integrated to explain the neuro-optometric rehabilitation concepts within the framework (Section 4).

The search strategy employed the following MeSH terms and keywords: Parkinson Disease [Mesh] OR “Parkinson’s disease” OR Parkinson*[tiab] OR “Parkinson disease”[tiab] OR Parkinsonism[tiab] OR “idiopathic parkinsonism”[tiab] AND “Ocular Motility Disorders”[Mesh] OR “Saccades”[Mesh] OR “Pursuit, Smooth”[Mesh] OR “Fixation, Ocular”[Mesh] OR “oculomotor dysfunction” OR “eye movement*” OR saccad* OR “smooth pursuit*” OR “fixational instability” OR nystagmus OR “ocular motility” OR strabismus OR diplopia OR convergence. A backward snowballing search strategy was applied to identify any qualifying research omitted by the initial search.

The literature selection process was designed to systematically populate the conceptual framework with relevant articles when a relationship was established between idiopathic PD and ocular motor deficits, involving at least one of the following domains: visual-sensory inputs (e.g., convergence insufficiency), visual-motor metrics (e.g., saccades, smooth pursuit, vergence), visual-perceptual processing (e.g., spatial localization), cognitive processing (e.g., attention), and downstream psychosocial and functional challenges affecting the activities of daily living (e.g., reading challenges). To ensure a focused synthesis within the broad thematic scope without compromising depth, data extraction applied the principle of data saturation by prioritizing representative articles that offered high-level evidence while omitting smaller studies. Articles on atypical parkinsonian syndromes (e.g., progressive supranuclear palsy) and those confounded by severe ocular pathologies and/or unrelated neurological conditions were excluded from this review to ensure the synthesized data reflects idiopathic PD pathology.

## 3. Sensorimotor Framework of Oculomotor Dysfunction

**Figure 1:**
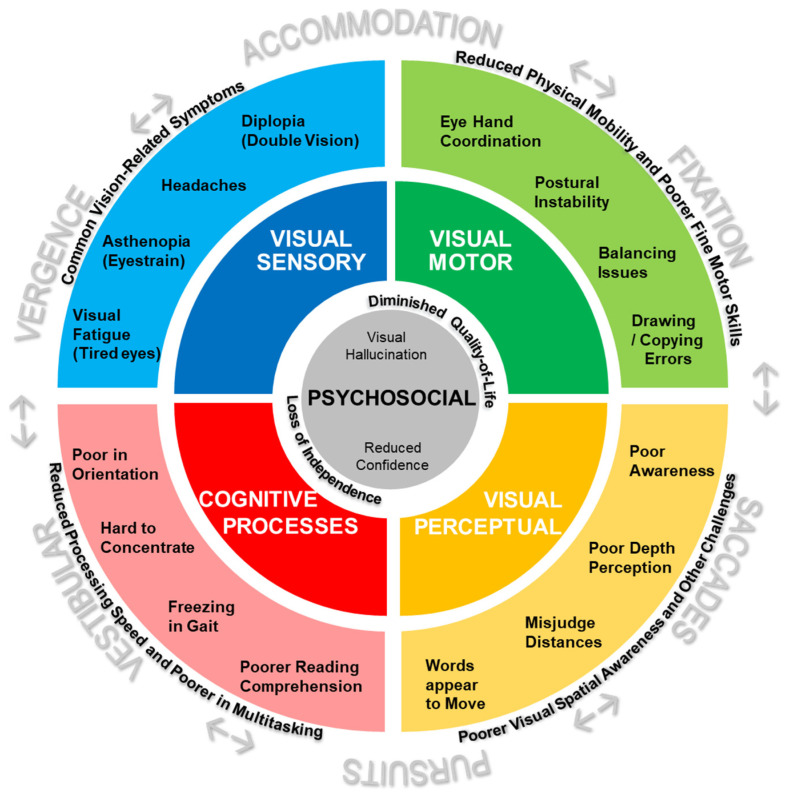
Sensorimotor framework of oculomotor dysfunction in Parkinson’s disease in relation to the vision-related symptoms and the challenges to activities of daily living.

The signs and symptoms of oculomotor dysfunction in patients with PD are largely dependent on its disease stage, subtype, comorbidities, age-related changes (e.g., cataract), medications [9,10], and compensatory mechanisms (e.g., head movements) when eye movements are impaired [11,12]. As part of the sensorimotor framework, there are five key implications to consider in patients with PD (Figure 1). This may be categorized in terms of visual-sensory, visual-motor, visual-perceptual, cognitive processing, and psychosocial challenges, and it explores how these challenges impact the patient’s symptoms, mobility, postural stability, spatial awareness, eye–hand coordination, processing speed, and quality-of-life. The framework’s outer circle depicts the neural elements essential to oculomotor function, including fixation, saccades, smooth pursuit, vergence, accommodation, and their integration with the vestibular system.

Firstly, there may be increased asthenopia and visual fatigue during prolonged near tasks [13,14], where greater visual demands tend to increase the level of difficulty for the patients. For example, 17% perceived words moving while reading [7], nearly 50% experienced headaches [15], and 18% complained about diplopia [7]. These symptoms typically arise when the brain struggles with the eyes focusing and alignment. One example of a vergence disorder is convergence insufficiency, which affects nearly 50% of patients with PD [13,14] A third of those with convergence insufficiency tend to experience diplopia in the distance due to hypertropia, exotropia, or esotropia [8], and nearly all patients with diplopia have convergence insufficiency [8]. While the symptoms may be partially attributed to bradyphrenia (slow cognitive processing speed) [16], the prolonged oculomotor execution is more likely attributable to the stress on the accommodative and vergence systems. This is because the visual system may be unable to sustain an accurate focus and alignment of the two eyes to perceive comfortably in order to stabilize the visual system and obtain a sharp retinal image [4]. This may in turn affect saccadic eye movements (to rapidly align the fovea from one place to another), and pursuit eye movements (to enable the patient to maintain a clear, steady, and continuous image on a slow-moving target).

Secondly, oculomotor dysfunction can affect tasks that require a higher cognitive demand [17,18]. For example, cognitive symptoms may increase due to the extra effort required to control eye movements, and this could drain cognitive resources, making it harder to concentrate, process information, or perform multi-tasking activities [19]. Since saccadic and pursuit eye movements are supposed to be conjugate with equal velocity and amplitudes, it requires the accommodative and vergence systems of the visual system to be tightly coupled in the process of fixation [4]. If the vergence and/or accommodative systems are poorly coordinated, it places additional stress on the visual system leading to unstable fixation, inaccurate eye movement, reduced stereopsis, and poor spatial localization. For example, the eyes may tend to make several small corrective saccades (e.g., saccadic intrusions and anti-saccades) instead of converging smoothly on a target [8]. Thus, patients with PD often produce hypometric saccades where the eyes undershoot the intended target instead of a single and complete movement [20], and it may take longer to initiate the movement (increased latency), and they may tend to slowdown (i.e., bradykinesia of saccades) [21]. This can affect reading efficiency, as it depends on stable fixation, accurate forward saccades, and minimal regressions [22,23]. Comprehension may decrease, and the patient may experience fatigue, feel distracted, or feel that “things are feeling worse” due to the extra cognitive effort needed to control eye movements. Approximately 30% of PD patients also experience “freezing of gait” in narrow spaces [24], difficulties in maintaining balance when walking or standing, and feeling uncomfortable when turning their head due to postural instability and oscillopsia [25] contributing to their symptoms and/or challenges.

Thirdly, challenges may arise when the brain is unable to perceive objects properly and/or interpret them correctly. For instance, 12.5% of PD patients report misjudging objects when walking [7], which may be related to poorer stereopsis, visuospatial awareness, balance, accuracy in scanning their environment for obstacles, and difficulty in judging distances. The neural correlates of poor stereopsis are related to reduced grey matter volume in the extrastriate visual areas [26]; motion perception impairments are related to deficits in the middle temporal area [27]. Poor pattern recognition and figure-ground discrimination are associated with reduced grey matter density in the superior parietal lobes [28], and difficulties in orientation judgements may be related to parietal lobe lesions [29]. This can affect their mobility, sense of orientation, and ability to navigate obstacles, as there involve visuo-perceptual processing, executive functioning, praxis and motor planning [25].

Fourthly, this can impact the patient’s activities of daily living especially if the tasks require fine visual motor control [1,19,25]. For example, they may experience difficulties with the clock drawing test, drawing houses, mental rotations, and copying complex figures (e.g., intersecting pentagons). Given that motor automaticity is often poor, there may be a greater tendency to rely more on visual information for motor and postural control [30], and this can interfere with motor planning and increase the chances of making errors (e.g., in cognitive tasks that involve matching objects). There may also be challenges in maintaining their balance due to poor integration with the vestibular and proprioceptive systems.

Fifthly, the cumulative effect of functional impairments can be debilitating, and this can lead to a cascade of negative effects, such as frustration, anxiety, depression, social isolation, and social withdrawal if these functional deficits remain unaddressed [31]. This can diminish quality-of-life, increase the risks of falls, reduce their confidence in walking especially in busy or unfamiliar environments, decrease independence, and lead to a greater reliance on caregivers. For example, lateral axial dystonia is a relatively common type of postural abnormality that makes it difficult for a person to walk [32]. These issues may be aggravated by other age-related conditions (e.g., cataracts) and uncorrected refractive errors (e.g., outdated prescription spectacles). The latter can also have an effect on vergence [19,25,33] which can disrupt the delicate balance and coordination required for efficient visual processing [19,25,33]. Thus, it is important to manage these conditions accordingly.

Sixthly, prominent abnormalities of oculomotor function may be early indicators of cognitive decline and it is known that some patients may progress to dementia [18]. Patients with oculomotor dysfunction tend to have a poorer prognosis [18], and recurrent complex visual hallucinations are more frequently experienced in those with dementia (89%) [24] than those without dementia (17–30%) [7,24]. However, the usage of speed-dependent mediators to predict speed-dependent outcomes introduces an issue of circular reasoning since both dopaminergic depletion and intrinsic oculomotor pathway lesions can disrupt the balance of excitatory and inhibitory signaling within the oculomotor network.

## 4. Neurorehabilitation of Oculomotor Dysfunction

Oculomotor dysfunction is an increasingly recognized feature of Parkinson’s disease [34,35,36] which is characterized by visual-sensory, visual-motor, visual-perceptual, cognitive processing, and psychosocial challenges. Given a more granular understanding of visual sensorimotor integrity, this framework for oculomotor dysfunction in PD enables the design of neurorehabilitation strategies by mapping the contribution of each involved neural element to the rehabilitation recommendations. This provides for a structured neurorehabilitation treatment plan in order to provide targeted interventions based on the distinct types of ocular motor deficits in fixation, saccadic eye movement, pursuit eye movement, vergence, accommodation, and their integration with the vestibular system—this approach is well-established in the field of neuro-optometric rehabilitation [4].

Clinical evaluation should move beyond a reliance on speed-dependent metrics that reflect generalized bradyphrenia. This is because eye movements are inherently tied to executive control networks rather than motor execution alone, and oculomotor dysfunction tends to occur independently of the patient’s general motor severity [2]. While it may be debatable that this is a result of a central cognitive bottleneck [16], the current evidence suggests that the deficits involved reflect a broader breakdown in the fronto-striatal and parietal networks, rather than a simple downstream degradation of the motor pathway [35,37,38,39]. Thus, a battery of specialized tests can be performed during a neuro-optometric evaluation, that is conducted in the context of functional tasks that put stress on oculomotor control and coordination, alongside an assessment of visual acuity, refraction, binocular vision, stereopsis, and ocular health [4,40]. Tests that provide speed- and non-speed-dependent metrics may be utilized. For example, thresholds of vergence and accommodation can be assessed under static and dynamic conditions. While eye-tracking technologies can quantify the deficit, this is not absolutely necessary as the technologies are currently not optimized to accurately capture information on vergence and accommodation [41]. Instead, the priority is to evaluate each neural element of the deficit in tandem with fixational stability and vestibular function [4].

**Figure 2:**
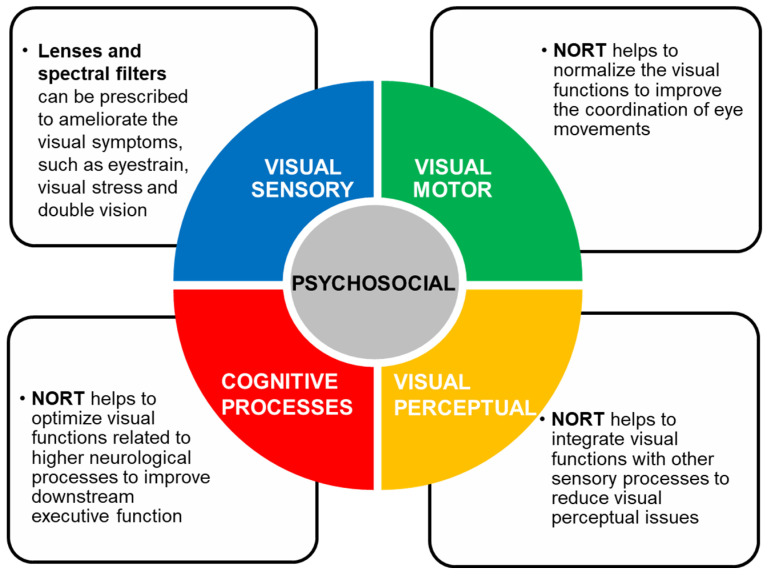
Neuro-optometric rehabilitation of oculomotor dysfunction in patients with Parkinson’s disease and the approaches in neuro-optometric rehabilitation therapy (NORT) to address the symptoms and challenges in activities of daily living.

Utilizing this framework, the neuro-optometric rehabilitation of oculomotor dysfunction is aimed firstly to ameliorate the symptoms, secondly to normalize the visual functions, thirdly to integrate each of these visual functions with one another, and fourthly to address the visual deficits in relation to higher neurological processes (Figure 2). This helps to restore eye-movement coordination, and to address poor spatial localization, which can in turn improve postural stability and reduce the risk of falls. Its core principles are similar to the internationally recognized INCOG 2.0 guidelines that were originally formulated to standardize cognitive rehabilitation following TBI [42], and these approaches can typically be achieved from a well-planned, structured neuro-optometric vision rehabilitation program that is customized according to the patient’s specific visual deficits. For example, intervention with neuro-optometric rehabilitation therapy (NORT) is usually targeted at restoring the patient’s control of fixation (to hold the gaze steady on a stationary target), which is crucially coupled with vergence (the eye’s ability to converge or diverge) and accommodation (the eye’s ability to rapidly adjust focus on an object) to maintain single vision. By leveraging goal-directed, individualized care, contextualized dual-task training, and metacognitive strategies [43], the neuro-optometric rehabilitation of oculomotor dysfunction in patients with PD is similarly designed to improve visual-sensory processing with ocular motor execution. Alternatively, patients who are less inclined to start NORT can explore compensatory approaches, such as relying on visual cues to help to improve walking patterns and overcome “freezing of gait” (e.g., lines on the floor), using head turns to assist with gaze shifts, or optimizing visual tasks with appropriate lighting and contrast adjustments.

Prior to commencing NORT, symptoms may be reduced with dopaminergic medications (e.g., levodopa) as prescribed by medical doctors, but non-motor symptoms like visual fatigue and asthenopia may be improved by updating any spectacle prescriptions with lifestyle modifications, such as exercises, nutrition, and rest. Although a loss of contrast sensitivity and color vision deficiency are less common in the early stages, they can become an issue when the disease advances [2,3]. Early losses of contrast sensitivity may tend to improve with medication, but it is less effective in more moderate to advanced stages [1]. If the symptoms fluctuate under “on” and “off” medication states, it may be possible to change the timing and/or dosage. Treatments for dry eyes [36], depression [44], anxiety [45], and sleep disturbances [46] may help to reduce symptoms. Proper task lighting can help to improve visual comfort, larger font sizes can help to reduce visual stress when reading, and the reduction of clutter can help to minimize visual distractions. However, it is important to note that these approaches generally do not address the root causes of oculomotor dysfunction, which must be carefully examined through a neuro-optometric evaluation.

Neurorehabilitation should begin with the provision of an optimal spectacle prescription to reduce symptoms and to meet the specific visual demand of the patient [13,19,25,33]. In cases of diplopia, prism lenses can be prescribed in spectacles to assist with fusion of images, especially during near visual tasks like reading [13]. Even in the absence of diplopia, the appropriate spectacle prescription can help to reduce asthenopia and visual stress; and reduce oculomotor-related postural deficits [32]. For example, yoked prism lenses can be prescribed to shift the visual field image to recalibrate the patient’s internal sense of “straight ahead” and correct a leaning posture and gait instability through prism adaptation [25,47,48]. This differs from base-in prism lenses which use opposing orientations to reduce convergence demand for reading and to eliminate diplopia [49], rather than for improving postural control and gait [25,47,48].

Neuro-optometric vision rehabilitation therapy focuses on treating specific functional vision deficits, and it is usually provided by optometrists with postgraduate fellowship or residency training in NORT (e.g., a Fellow of the Neuro Optometric Rehabilitation Association [FNORA], a Fellow of Optometric Vision Development and Rehabilitation [FOVDR], a Fellow of the Australasian College of Behavioral Optometrists [FACBO]). Specialized treatment plans are typically necessary for patients to reach successful outcomes, and regular follow-ups are crucial for treatment monitoring. By improving stereopsis, reducing saccadic hypometria, improving smooth pursuit eye movements, and integrating these visual functions with the vestibular system, functional vision deficits are reduced with improvements to visuomotor control, posture, and balance. Treatment plans should also take into consideration the higher neurological processes, including the ventral stream (e.g., V1 through areas V2 and V4 to the inferior temporal cortex) that is responsible for object identification, and the dorsal stream (e.g., V2 and V3 to the superior temporal cortex and the parietal cortex) which processes spatial relations and movement [25]. It is important to keep the spectacle prescriptions up-to-date, and modify treatment strategies as needed. In addition, care can improve visual guidance skills, which is critical for the patient to orientate and navigate in crowded environments, to perform activities of daily living.

The treatment plans in NORT can be broadly conceptualized using the Skeffington’s four circles [50], a well-established concept of behavioral optometry to improve coordination of the visual system in relation to the other senses and the body, namely: (1) the volume of space (i.e., centering), (2) the process of identification, (3) gravity and the sense of orientation (i.e., anti-gravity), and (4) the ability to share information (speech-auditory). For example, the centering of attention enables patients to use vergence to assess the difference between their own internal perception of visual space and the actual physical space (i.e., “where is it”), whereas identification involves the process of accommodation and recognition (i.e., “what is it”) [50]. Skeffington’s concepts of centering and identification are akin to the concepts of peripheral and central vision—while doing near-centered work, patients with exophoric postures tend to overemphasize the latter and reduce peripheral awareness [50]. These functions are mediated by the dorsal and ventral streams in the brain respectively [51], and NORT can be implemented to improve processing by integrating both the broader visuo-spatial information in the periphery and the detailed central vision [25].

To improve the sense of orientation, treatment plans can involve a strong interplay between the visual, vestibular, and proprioception systems, where movements can be considered as successive changes in relation to gravity (“anti-gravity”), and posture can be considered the relative position of the physical parts of the body in relation to each other and relative to gravity [25]. For example, some therapeutic activities involve the use of a balance beam and movement (e.g., walking a balance beam or using a trampoline) to encourage the visual system to work with the vestibular system in maintaining balance and spatial awareness [25]. Thus, NORT can help to integrate these systems to improve visual guidance skills. In addition, the integration of visual processing with speech-auditory processing can improve visual attention, sequencing, and the ability to organize visual information, which indirectly supports auditory processing and communication [25]. For example, a metronome can be used with prescribed vision activities, or the matching of visual cues with auditory instructions to train the brain to process both sensory inputs simultaneously.

While neurologists may flag its initial symptoms and ophthalmologists or neuro-ophthalmologists may rule out structural abnormalities and eye diseases, a detailed evaluation of oculomotor dysfunction is often omitted under time constraints in acute hospitals [52]. However, some of the earliest neurological changes of Parkinson’s disease may be observed from an optical coherent tomography (OCT) of the eye, such as thinning of the retinal nerve fiber layer and the macular ganglion cell-inner plexiform layer [53], which can potentially serve as biomarkers for the diagnosis and progression of Parkinson’s disease. Conversely, there may be a higher chance for physical therapists (physiotherapists), occupational therapists, and optometrists in the community to recognize oculomotor dysfunction since rehabilitation tends to be goal-oriented in improving movement, balance, and independence [54]. For example, it is more likely for the rehabilitation team to observe a patient’s struggle to track a moving target during a balance exercise, or difficulty in performing fine motor tasks or reading. Furthermore, medications such as anticholinergics (e.g., benzhexol and diphenhydramine) and antivirals (e.g., amantadine) can affect accommodation [13], benzhexol can cause angle closure of the eye [9], and abnormalities in blinking, such as a reduced blinking rate and blepharospasm, may lead to dry eyes [9]. Therefore, these problems need to be investigated by optometrists and ophthalmologists.

Should oculomotor dysfunction be used as a predictor of future dementia in patients with PD [18], caution must be exercised as there is still a debate surrounding central cognitive bottlenecks versus localized motor deficits contributing to dementia. To avoid falling into circular reasoning, future research should employ experimental designs that explicitly decouple temporal constraints from task performance. Theoretically, this can be achieved by establishing an independent cognitive baseline that bypasses the oculomotor system, alongside an assessment of visual sensorimotor integrity based on neuro-optometric approaches. If eye-tracking technologies are used in this situation, non-speed dependent metrics, such as the amplitude of saccadic dysmetria and the frequency of intrusive catch-up saccades during smooth pursuit, will be preferred, rather than measuring the velocity and latency of the saccadic or pursuit eye movements.

## 5. Conclusions

This review has characterized oculomotor dysfunction in patients with PD based on the distinct types of ocular motor deficits and their corresponding impact on the patient’s symptoms, visual perception, activities of daily living, and quality-of-life. The findings from this framework suggest that clinical evaluation and neurorehabilitation should move beyond speed-dependent metrics, and focus on specific non-speed-dependent ocular motor deficits. Ultimately, successful neurorehabilitation relies on targeting specific deficits in saccades, pursuits, vergence, and accommodation, while ensuring integration with fixational and vestibular systems. By fostering interdisciplinary collaboration, healthcare professionals can take proactive steps to address the vision-related challenges faced by these patients and help them to maintain optimal visual function and their quality-of-life.

## Data Availability

The original contributions presented in this study are included in the article. Further inquiries can be directed to the corresponding author.
